# Atypical diabetes with spontaneous remission associated with systemic lupus erythematosus in an adolescent girl of African ancestry, a case report

**DOI:** 10.1186/s12902-023-01478-0

**Published:** 2023-10-20

**Authors:** Fanny Luterbacher, Jean-Louis Blouin, Valerie M. Schwitzgebel

**Affiliations:** 1 Pediatric Endocrinology and Diabetology, Department of Pediatrics, Gynecology and Obstetrics, University Hospitals of Geneva, Rue Willy-Donzé 6, 1205 Geneva, Switzerland; 2 Department of Genetic Medicine and Development, Faculty of Medicine, University of Geneva, 1211 Geneva, Switzerland; 3 Department of Diagnostics, University Hospitals of Geneva, 1211 Geneva, Switzerland; 4 Diabetes Center of the Faculty of Medicine, University of Geneva, 1211 Geneva, Switzerland

**Keywords:** Case report, Flatbush diabetes, Monogenic diabetes, C-peptide, Beta cell, Diabetic ketoacidosis, Autoantibodies, Autoimmune

## Abstract

**Background:**

New-onset diabetes in youth encompasses type 1 diabetes, type 2 diabetes, monogenic diabetes, and rarer subtypes like Type B insulin resistance syndrome and ketosis-prone atypical diabetes in African populations. Some cases defy classification, posing management challenges. Here, we present a case of a unique, reversible diabetes subtype.

**Case presentation:**

We describe an adolescent African girl recently diagnosed with systemic lupus erythematosus. At age 15, she presented with ketoacidosis, HbA1c of 108.7 mmol/mol (12.1%), and positive anti-insulin antibodies. Initially diagnosed with type 1 diabetes, insulin was prescribed. Due to the presence of obesity and signs of insulin resistance, we added metformin. Concurrently, she received treatment for lupus with hydroxychloroquine, mycophenolate mofetil, and prednisone. After discharge, she stopped insulin due to cultural beliefs. Five months later, her glycemia and HbA1c normalized (37 mmol/mol or 5.5%) without insulin, despite corticosteroid therapy and weight gain. Autoantibodies normalized, and lupus activity decreased. Genetic testing for monogenic diabetes was negative, and the type 1 genetic risk score was exceptionally low.

**Conclusions:**

We present a complex, reversible diabetes subtype. Features suggest an autoimmune origin, possibly influenced by overlapping HLA risk haplotypes with lupus. Lupus treatment or immunomodulation may have impacted diabetes remission. Ancestry-tailored genetic risk scores are currently designed to improve diagnostic accuracy.

**Supplementary Information:**

The online version contains supplementary material available at 10.1186/s12902-023-01478-0.

## Background

New-onset diabetes in youth can fall into three categories: type 1 diabetes (T1D), type 2 diabetes (T2D), or monogenic diabetes (MD) [1]. This classification is based on underlying disease mechanisms, dictates treatment, and predicts outcomes. Type 1 diabetes is an autoimmune disease leading to the destruction of beta cells and is characterized by circulating anti-diabetes antibodies. As T1D results in absolute insulin deficiency, insulin replacement therapy is mandatory. The natural progression of the disease often includes a honeymoon phase after diagnosis, where patients benefit from improvement in their glycemic controls with reduced exogenous insulin needs [2, 3]. Unfortunately, insulin requirements increase after that period, and complete remission is unlikely as autoimmunity persists over time.

Ketosis-prone atypical diabetes (KPD) is a heterogenous type of diabetes found mainly in the African population. Patients usually present with ketosis or ketoacidosis requiring insulin treatment, but autoimmune markers often remain negative [4, 5]. The American Diabetes Association classifies this diabetes form as strongly inherited idiopathic T1D but not HLA-associated [6]. The cause of beta-cell dysfunction remains unknown.

Type 2 diabetes is an increasing health concern in pediatrics, with the highest incidence among American Indians and African Americans [7, 8]. So, ethnicity is a risk factor.

Monogenic diabetes involves the mutation of a single gene. The gene defects provoke a reduction of the number of beta cells or a disruption of the beta-cell function [9, 10]. Monogenic diabetes accounts for 3 to 4% of diabetes in youth [11].

Type B insulin resistance syndrome (TBIR) is caused by autoantibodies binding antagonistically to the insulin receptor preventing its normal function [12]. This condition is also more prevalent among people of African descent. It is a rare disorder often combined with other autoimmune diseases [13], such as systemic lupus erythematosus (SLE). In TBIR, insulin needs are significantly increased due to insulin resistance, and TBIR does not classically present with ketoacidosis. Clinically, acanthosis nigricans and hyperandrogenism are common [14]. Spontaneous remission is described, but mortality is high due to hyperglycemia evolving towards hypoglycemia. A positive response to glucocorticoid therapy has been reported [15]. In this case report, we illustrate the case of a patient with an atypical diabetes that does not fit into the classical clinical and biological characteristics of the different subtypes of diabetes known to date.

## Case presentation

The adolescent, a 14-year-old patient of Congolese origin, was known for obesity (BMI 31 kg/m^2^) and a diagnosis of SLE (anti-double-stranded DNA antibody 93 UI/ml) three months before diabetes onset. She was treated with hydroxychloroquine (4.8 mg/kg/d), mycophenolate mofetil (3.05 mg/kg/d) and prednisone (0.75 mg/kg/d) (Table 1). The family history was negative for diabetes or maternal gestational diabetes. She developed features of T1D, including polyuria and polydipsia, with a weight loss of 11 kg (from 99 to 88 kg) over 2.5 weeks. At admission she had ketoacidosis (pH 7.26, PCO2 2.9 kPa, glucose 29 mmol/l, lactate 2 mmol/l, bicarbonate 10.2 mmol/l, base excess -15.5 mmol/l, ketones (beta-hydroxybutyrate 6.5 mmol/l) (Table 2).

**Table 1:** Treatments at admission, discharge, and follow-up

Treatments	At admission	At discharge	Five months after diabetes diagnosis	14 months after diabetes diagnosis
Prednisone (mg/d)	30	30	5	40
Insulin (U/kg/d)	-	1.49	-	-
Metformin (mg)	-	2 × 850	-	-
Mycophenolate mofetil (g)	3 × 1	3 × 1	3 × 1	3 × 1
Hydroxy-chloroquine (mg)	400	400	400	400

**Table 2:** Clinical characteristics at admission and follow-up

	**At admission**	**Five months after diabetes diagnosis in remission**
Age (years)	15	15
Weight (kg)	88	96
Height (m)	1.8	1.8
BMI (kg/m2)	27.2	29.6
Insulin autoantibody (U/ml) N < 2.4	5.12	0.6
IA2 autoantibody (IU/ml) N < 15	-	< 15
GAD autoantibody (IU/ml) N < 5	< 4	< 4
ZnT8 autoantibody (IU/ml) N < 15	< 1.2	4.4
Hb1Ac (mmol/mol)	108.6	37
Hb1Ac (%)	12.1	5.5
Fasting glycemia (mmol/l) N 3–6.5	10.1	5
Fasting Insulin (mUI/l) N 2.6–24.9	-	46.3
HOMA N < 4	-	10.3
Ketones (mmol/l) N < 0.5	6.5	0
C-Peptide (pmol/l) N 370–1′470	532	1334
Fructosamine (μmol/l) N 100–285	775	-
Total Cholesterol (mmol/l) N 2.91–5.36	5.9	-
Triglycerides (mmol/l) N 0.54–2.47	1.34	-
TSH (mUI/l) N 0.27–4.2	0.758	-

*N* normal value

**Fig. 1:**
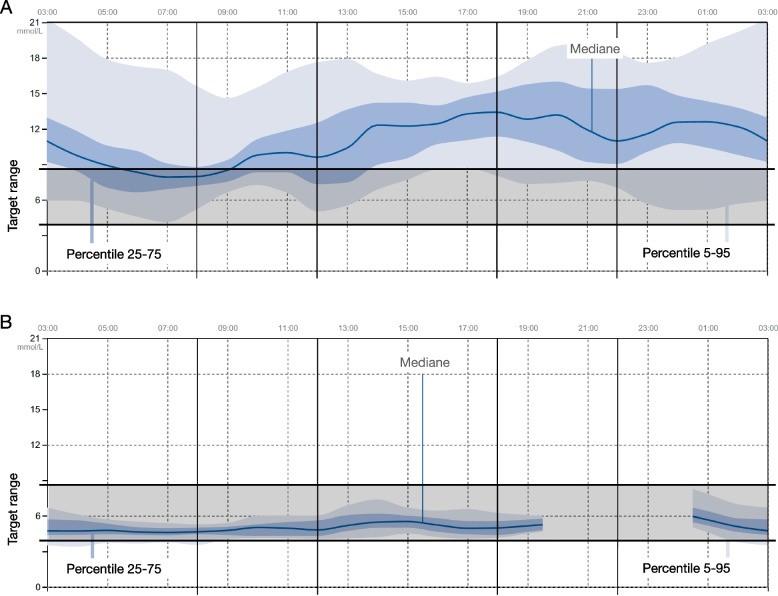
Flash glucose monitoring 1. Glucose sensor data. **A** While hospitalized at the onset of diabetes, despite receiving a total daily insulin dose of 1.39 IU/kg/day, the patient experienced prolonged periods of hyperglycemia (greater than 10 mmol/l). **B** Six months later, without any anti-diabetic treatment, glycemic levels were successfully normalized

We initiated classical management of ketoacidosis by intravenous followed by subcutaneous insulin therapy. Glucose-sensor downloads depict the glucose levels during hospitalization (Fig. 1A). After therapeutic education of the girl and her family, she was discharged from the hospital with insulin pump therapy and metformin (850 mg 2x/d) due to high insulin needs (1.49U/kg/d). Subsequent check-ups in the ambulatory care clinic showed an insufficient control of glycemia with an HbA1c of 64 mmol/mol (8.0%). Insulin requirements were at 1.38 UI/kg/d one month after diagnosis. After that, insulin needs slowly declined, and the patient stopped insulin and metformin treatment at the same time two months after the diabetes diagnosis. Three months later, we noticed a normalization of the HbA1c at 37 mmol/mol (5.5%) and an increase in C-peptide levels up to 1334 pmol/l without any anti-diabetic treatment (Table 1, Fig. 1B). Twenty months after the initial diabetes onset, while still on prednisone, the HbA1c was at 40 mmol/mol (5.8%).

### Genetic results

We performed a genetic analysis in search of monogenic diabetes. We detected no pathogenic or likely pathogenic variant in the analyzed genes, including *GCK, HNF1A, HNF1B, HNF4, INSR, and APPL1* genes or the mitochondrial variant m.3243A > G. The type 1 genetic risk score (GRS) to assess T1D susceptibility was below the 1^st^ centile [16]. The low score does, however, not exclude T1D entirely.

## Research design and methods

### Exome sequencing

We performed the extraction of DNA from venous blood. We executed exome capture and sequencing (Twist Human Core Exome capture kit + RefSeq_V1 EF Multiplex, Illumina NextSeq500 sequencer) according to the manufacturer’s instructions. We used the locally developed bioinformatics pipeline for sequence analysis. The exome sequencing coverage was 10 × 99.2% and 20 × 99.1%. PCR and Sanger sequencing was done for variant search in the promoter of *HNF1A* and *HNF4A* genes. We did MLPA for the exclusion of deletion or extended duplication at one or more exons of *the GCK, HNF1A, HNF1B*, and *HNF4A* genes (Kit MRC-Holland Salsa P241_E1) and PCR HRM (hybridization probe melting curve) for the search of the mitochondrial mutation m.3243A > G (Table 3).

### Type 1 genetic risk score

The GRS involves genotyping of common genetic variants that have been found to contribute to T1D susceptibility [16, 17]. The score was calculated as previously published by Oram et al. [16].

## Discussions and conclusions

Our patient's presentation with ketoacidosis and mildly positive anti-insulin antibodies in the context of SLE raises intriguing clinical questions. The complete remission of diabetes within a few months following diagnosis, notably without associated weight loss, is a rare and unexpected occurrence, particularly considering the presence of ketoacidosis and signs of autoimmunity. While a few analogous cases have been documented in adults, our patient represents a unique pediatric presentation [12, 18].

The atypical course of diabetes remission challenges conventional T1D expectations. Notably, the patient experienced weight gain during remission while receiving glucocorticoid therapy, arguing against a diagnosis of T2D or steroid-induced diabetes. Furthermore, comprehensive exome analysis ruled out monogenic diabetes. One conceivable diagnosis is TBIR, a condition predominantly affecting females and African Americans. TBIR often co-occurs with other autoimmune diseases, including SLE [19]. It is characterized by autoantibodies targeting the insulin receptor, leading to a heterogeneous metabolic syndrome, frequently observed in the African American population. Approximately one-third of TBIR cases witness the spontaneous disappearance of antibodies alongside the correction of metabolic dysfunction [14]. However, the remission of diabetes following treatment with glucocorticoids [15], cyclophosphamide, rituximab [19], or mycophenolate, as observed in our patient, has not been reported in TBIR. Additionally, the patient's maximum daily insulin requirement (146 U/day) does not align with typical TBIR insulin needs, exceeding 750 U/day, up to 18,000 U/day [19].

We posit that disease activities and severity can be interconnected in patients with multiple autoimmune disorders. SLE is characterized by substantial gene expression alterations, affecting a wide array of genes across various tissues [20]. Such gene expression disruptions can propagate abnormal regulation in multiple genes [21], potentially influencing beta-cell function and insulin production. Recent research also suggests a role for specific immune signaling factors, such as interleukin-21 (IL-21) and follicular T helper cells, in driving immune responses and antibody production in autoimmune diseases. The association between IL-21 production and the development of diseases like SLE and T1D has been documented [22]. It is possible that simultaneous treatment for SLE influenced the remission of diabetes in our patient, as her therapy with prednisone and mycophenolate mofetil may have suppressed antibody-producing plasma cells and T-lymphocytic responses.

Despite the clinical and laboratory findings, pinpointing the precise origin of diabetes remains challenging due to the limitations of existing markers (antibodies, C-peptide) [16]. Novel diagnostic tools tailored to diverse ethnic backgrounds are essential. The GRS we employed, while informative, lacks validation for individuals of African ancestry and thus proved unreliable for diabetes classification. Emerging ancestry-specific GRS are currently under evaluation [23] and hold promise for distinguishing between different diabetes forms in ethnically diverse populations [24].

In conclusion, our case presents a compelling instance of a complex and reversible diabetes subtype. Several distinctive features, including the presence of albeit faint autoantibodies and the coexistence of SLE, point towards an autoimmune origin. Notably, SLE and T1D share HLA disease risk haplotypes [25], adding complexity to the diagnostic puzzle. Despite our efforts, the polygenic risk score, unsuited for those of African ancestry, did not align with a T1D diagnosis. We propose the intriguing possibility that the immunomodulatory treatment administered for SLE may have halted the development of T1D. While T2D appears unlikely given the remission during weight gain and glucocorticoid therapy, TBIR, often associated with SLE, remains a less probable explanation due to the incongruence in insulin requirements. Monogenic diabetes, at least among currently known genes, has been ruled out.

This atypical form of KPD warrants continued follow-up and ongoing research to unravel the underlying biological mechanisms governing disease development. As we strive to enhance our understanding of such complex cases, new diagnostic tools, and tailored GRSs, especially for diverse populations, hold promise for achieving more precise and accurate diagnoses in the future.

### Supplementary Information

**Additional file 1: Table 3.** List of genes and accessions.

## Data Availability

Supplementary information: Table 3. The data used to support the findings of this study are available from the corresponding author upon request. Sequences are available in the zenodo database under accession number 10.5281/zenodo.7463814. (https://zenodo.org/record/7463814#.Y6HAlLLMKgZ).

## Abbreviations

T1D: Type 1 diabetes
T2D: Type 2 diabetes
MD: Monogenic diabetes
SLE: Systemic lupus erythematosus
TBIR: Type B insulin resistance
KPD: Ketosis-prone diabetes
HbA1c: Hemoglobin A1c
GRS: Genetic risk score
