# Supplementary material for: Atypical diabetes with spontaneous remission associated with systemic lupus erythematosus in an adolescent girl of African ancestry, a case report
Source: BMC Endocr Disord. 2023 Oct 20;23:228. doi: 10.1186/s12902-023-01478-0 (PMC10588024; doi:10.1186/s12902-023-01478-0)
Supplement: Supplementary file 1 — Additional file 1: Table 3. List of genes and accessions. [file 12902_2023_1478_MOESM1_ESM.docx]

Appendix

Table 3. List of genes and accessions

| **Gene name** | **RefSeq accession number (Genbank)** | **Chromos. Location** |
| --- | --- | --- |
| **Genes already in the design 04818-1448461538** |  |  |
| ***ABCC8*** | [NM_000352](http://www.ncbi.nlm.nih.gov/nuccore/NM_000352) | Chr.11 |
| ***BLK*** | [NM_001715](http://www.ncbi.nlm.nih.gov/nuccore/NM_001715) | Chr.8 |
| ***CACNA1D*** | NM_000720.3 | Chr.3 |
| ***CEL*** | [NM_001807](http://www.ncbi.nlm.nih.gov/nuccore/NM_001807) | Chr.9 |
| ***CISD2*** | NM_001008388 | Chr.4 |
| ***DNAJC3*** | NM_006260 | Chr.13 |
| ***DUT*** | NM_001025248.1 | Chr.15 |
| ***EIF2AK3*** | [NM_004836](http://www.ncbi.nlm.nih.gov/nuccore/NM_004836) | Chr.2 |
| ***FOXP3*** | [NM_014009](http://www.ncbi.nlm.nih.gov/nuccore/NM_014009) | Chr.X |
| ***GATA4*** | [NM_002052](http://www.ncbi.nlm.nih.gov/nuccore/NM_002052) | Chr.8 |
| ***GATA6*** | [NM_005257](http://www.ncbi.nlm.nih.gov/nuccore/NM_005257) | Chr.18 |
| ***GCK*** | [NM_000162](http://www.ncbi.nlm.nih.gov/nuccore/NM_000162) | Chr.7 |
| ***GLIS3*** | [NM_152629](http://www.ncbi.nlm.nih.gov/nuccore/NM_152629) | Chr.9 |
| ***HNF1A*** | [NM_000545](http://www.ncbi.nlm.nih.gov/nuccore/NM_000545) | Chr.12 |
| ***HNF1B*** | [NM_000458](http://www.ncbi.nlm.nih.gov/nuccore/NM_000458) | Chr.17 |
| ***HNF4A*** | [NM_000457](http://www.ncbi.nlm.nih.gov/nuccore/NM_000457) | Chr.20 |
| ***IER3IP1*** | [NM_016097](http://www.ncbi.nlm.nih.gov/nuccore/NM_016097) | Chr.18 |
| ***INS*** | [NM_000207](http://www.ncbi.nlm.nih.gov/nuccore/NM_000207) | Chr.11 |
| ***KCNJ11*** | [NM_000525](http://www.ncbi.nlm.nih.gov/nuccore/NM_000525) | Chr.11 |
| ***KCNQ1*** | NM_000218.2 | Chr.11 |
| ***KDM6A*** | NM_021140 | Chr.X |
| ***KLF11*** | [NM_003597](http://www.ncbi.nlm.nih.gov/nuccore/NM_003597) | Chr.2 |
| ***KMT2D*** | NM_003482 | Chr.12 |
| ***LRBA*** | NM_001199282 | Chr.4 |
| ***MNX1*** | [NM_005515](http://www.ncbi.nlm.nih.gov/nuccore/NM_005515) | Chr.7 |
| ***NEUROD1*** | [NM_002500](http://www.ncbi.nlm.nih.gov/nuccore/NM_002500) | Chr.2 |
| ***NEUROG3*** | NM_020999 | Chr.10 |
| ***NKX2-2*** | [NM_002509](http://www.ncbi.nlm.nih.gov/nuccore/NM_002509) | Chr.20 |
| ***PAX4*** | [NM_006193](http://www.ncbi.nlm.nih.gov/nuccore/NM_006193) | Chr.7 |
| ***PAX6*** | [NM_000280](http://www.ncbi.nlm.nih.gov/nuccore/NM_000280) | Chr.11 |
| ***PCBD1*** | NM_000281 | Chr.10 |
| ***PDX1*** | [NM_000209](http://www.ncbi.nlm.nih.gov/nuccore/NM_000209) | Chr.13 |
| ***PLAGL1*** | [NM_001080954](http://www.ncbi.nlm.nih.gov/nuccore/NM_001080954) | Chr.6 |
| ***PPP1R15B*** | NM_032833 | Chr.1 |
| ***PTF1A*** | NM_178161 | Chr.10 |
| ***RAP1A*** | NM_001010935 | Chr.1 |
| ***RAP1B*** | NM_015646 | Chr.12 |
| ***RFX6*** | [NM_173560](http://www.ncbi.nlm.nih.gov/nuccore/NM_173560) | Chr.6 |
| ***SIRT1*** | NM_012238 | Chr.10 |
| ***SLC19A2*** | [NM_006996](http://www.ncbi.nlm.nih.gov/nuccore/NM_006996) | Chr.1 |
| ***SLC2A2*** | [NM_000340](http://www.ncbi.nlm.nih.gov/nuccore/NM_000340) | Chr.3 |
| ***STARD10*** | NM_006645.2 | Chr.11 |
| ***STAT3*** | [NM_139276](http://www.ncbi.nlm.nih.gov/nuccore/NM_139276) | Chr.17 |
| ***TMRT10A*** | NM_152292 | Chr.4 |
| ***WFS1*** | [NM_006005](http://www.ncbi.nlm.nih.gov/nuccore/NM_006005) | Chr.4 |
| ***ZPF57*** | [NM_001109809](http://www.ncbi.nlm.nih.gov/nuccore/NM_001109809) | Chr.6 |
